# Interaction Between Florfenicol and Doxycycline Involving Cytochrome P450 3A in Goats (*Capra hricus*)

**DOI:** 10.3389/fvets.2021.759716

**Published:** 2021-10-18

**Authors:** Xiaojing Wang, Yaqin Yang, María-Aránzazu Martínez, Marta Martínez, Bernardo Lopez-Torres, María-Rosa Martínez-Larrañaga, Xu Wang, Arturo Anadón, Irma Ares

**Affiliations:** ^1^National Reference Laboratory of Veterinary Drug Residues (HZAU) and MAO Key Laboratory for Detection of Veterinary Drug Residues, Huazhong Agricultural University, Wuhan, China; ^2^MOA Laboratory for Risk Assessment of Quality and Safety of Livestock and Poultry Products, Wuhan, China; ^3^Department of Pharmacology and Toxicology, Faculty of Veterinary Medicine, Universidad Complutense de Madrid, Madrid, Spain

**Keywords:** florfenicol, florfenicol amine, doxycycline, metabolism, interaction, CYP3A, residue depletion study

## Abstract

When two drugs are combined, drug-drug interactions (DDI) often occur. Metabolic DDI usually occur due to inhibition of the metabolism of one drug by the other. This leads to an increase in the plasma concentration of the drug whose metabolism is inhibited. The objective of this research study was to verify the DDI risk of two antibacterial, florfenicol (FF) and doxycycline (DOX) due to metabolism. Because food containing residues of any pharmacologically active substance could potentially constitute a public health hazard, we selected a food producing animal, goat, goat liver microsomes and recombinant metabolic enzymes, for *in vivo* and *in vitro* metabolism studies. *In vitro* experiments showed that CYP3A was the key enzyme subfamily in FF metabolism, DOX slowed down FF metabolism and R440 was possibly the key amino acid in the metabolic interaction between FF and DOX. *In vivo* studies in the goats showed that DOX inhibited up-regulation of CYP3A24 gene expression produced by FF; in liver and kidney, DOX slightly slowed down FF metabolism. Quantitative prediction of DDI risk suggest that when DOX is used in combination with FF in veterinary medicine, may result in a clinical significant increase of FF plasma and tissue concentrations, resulting a prevalence of harmful tissue residues of medicinal products in the food chain. Through our experimentation, when DOX is used in combination with FF, the withdrawal period of FF in the kidney was extended by 1 day. Otherwise, an appropriate withdrawal period (20 days) of FF was established for FF and DOX combined use to ensure that the animal can be safely slaughtered for food.

## Introduction

Florfenicol (FF), 2-dichloro-N-{(1R,2S)-3-fluoro-1-hydroxy-1-4-(methylsulfonyl) phenyl] propan-2-yl} acetamide, is a synthetic, broad-spectrum, primarily bacteriostatic antibiotic, of choice for the treatment of pneumonia and associated respiratory infections in livestock (1, 2). It is indicated in goats for the treatment of respiratory infections caused by *Mannheimia haemolytica, Pasteurella multocida*, and *Histophilus somni* associated with pyrexia (3, 4). The total FF residue in the tissue is calculated as the sum of FF and florfenicol amine (FFA). According to the European Union Regulation (EU) (5), the maximum residue limits (MRLs) of FF in goats are as follows: muscle (200 μg/kg), liver (3,000 μg/kg) and kidney (300 μg/kg). The MRL represents one of several standard options for risk managers to limit the presence of unwanted substances.

One of appropriate and suitable methods that would increase the effectiveness of chemotherapy of bacterial infections is a rational use of a combination of antimicrobial agents. Combination of antimicrobial agents is often presented as one of the few remaining effective strategies for the treatment of clinical diseases for which standard treatments have become ineffective. When two or more drugs are combined, the combinational effect can be defined as synergism, no interaction, or antagonism. Doxycycline (DOX), α-6-deoxy-5-hydroxytetracycline, a “second-generation” tetracycline, is also a primarily bacteriostatic antibiotic indicated in gastrointestinal and respiratory tract infections (6, 7). The combined use of FF and DOX has a synergistic or additive effect, both are usually combined to improve their efficacy. A study was conducted to study *in vitro* (*P. multocida* isolates) synergism of FF with DOX. The results reveal that synergistic interactions were observed in 15% of the tested isolates for FF + DOX. Consideration of synergism plus fractional inhibitory concentration index produced higher overall percentages of FF + DOX (69%) combination (8).

However, when more than one drug is used concurrently in combination therapy, drug-drug interactions (DDI) may occur, especially if their metabolism involves overlapping mechanisms (9). Cytochrome P450 (CYP450) enzyme-based drug metabolism is a key factor in DDI (10). Existing studies have shown that FF metabolism in rabbits and chickens is affected by CYP3A, and when P450 enzyme substrates, inhibitors or inducers are added, the drugs may interact and cause adverse effects (11, 12). In addition, studies have shown that DOX can inhibit the metabolism of quinine to 3-OH quinine via CYP3A (13). That is, DOX is likely to have an inhibitory effect on CYP3A. However, the possibility of DDI between DOX and FF under the influence of CYP3A has not yet been investigated.

Studies on the pharmacokinetics of FF in goats are relatively extensive, but there are few studies on the detection of FF tissue residues and the recommended withdrawal period (14, 15). The “withdrawal period” means the minimum period between the last administration of a veterinary medicinal product to an animal and the production of foodstuffs from that animal which under normal conditions of use is necessary to ensure that such foodstuffs do not contain residues in quantities harmful to public health (16).

With the gradual expansion of meat goat herds, goat meat has become an increasingly important animal-derived food, and its tissue residues have also received widespread attention (17). In this study, *in vitro* and *in vivo* experiments were carried out in goats to evaluate the DDI produced when DOX and FF are combined.

## Materials and Methods

### Bacteria and Plasmid

*Escherichia coli* BL21 (DE3) and *E. coli* DH5α were purchased from Vazyme (Nanjing, China). Plasmid pET-28a was obtained from the National Reference Laboratory of Veterinary Drug Residues (HZAU), Wuhan, China.

### Experimental Methods

#### Goat Liver Microsomes Experiments

Goat liver microsomes were purchased from PrimeTox Bio-medical Technology Co. LTD (Wuhan, China). The protein concentration was 20 mg/kg and the activity of the microsomes was appropriate. To determine the key metabolic enzymes of FF metabolism, we used six CYP450 inhibitors: ketoconazole (CYP3A4), diosmetin (CYP1A), quinidine (CYP2D6), fomepizole (CYP2E1), methoxsalen (CYP2A6), and sulfaphenazole (CYP2C9) were co-incubated with FF in goat liver microsomes. In order to study the effect of DOX on FF, we divided the experiment into a single group of florfenicol and a combined group of florfenicol and doxycycline. Both of these two combinations were incubated with goat liver microsomes. The production of FFA was determined by liquid chromatography tandem mass spectrometry (LC-MS/MS). Each reaction was performed in triplicate.

#### Cloning of the CYP3A24 Gene

According to the mRNA sequence of goat CYP3A24, specific primers (forward: 5′-cagcaaatgggtcgcggatccATGGAGCTAATCCCAAGCTTTTC-3′ and reverse: 5′-gtggtggtggtggtgctcgagGGCTCCACTTATGGTTCCATCTC-3′) were designed containing restriction enzyme sites *BamH* I and *Xho* I to amplify the coding sequence of CYP3A24. The goat CYP3A24 gene fragment was obtained by polymerase chain reaction (PCR) and ligated into the expression plasmid pET-28a. The ligated plasmid pET-28a-CYP3A24 was used to transformed *E. coli* DH5α. Isolated transformant was cultured and the recombinant plasmid was confirmed by sequencing.

#### Homology Modeling and Molecular Docking

Using the homology modeling phantom of the Sybyl-X2.0 software (Tripos, USA), the 3D structure of goat CYP3A24 was obtained based on the crystal structure of the human CYP3A4 protein (PDB ID: 4D7D). Sybyl-X2.0 was also used for molecular docking of the CYP3A24 protein with FF and DOX to obtain the key amino acids (AA) involved in the formation of hydrogen bonds between the protein and each drug (AA within 5Å of the active pocket are considered possible AA related to affinity) (18).

#### Site-Directed Mutagenesis

The steps for site-directed mutagenesis were as follows: First, the recombinant plasmid pET-28a-CYP3A24 was used as a template to obtain the required mutant plasmid PCR, and the primers used to introduce the mutation are shown in Table 1. Second, the mutant plasmid was digested with the restriction enzyme *Dpn* I and then used to transform *E. coli* DH5α cells (19). Third, the mutant plasmid was transformed into *E. coli* BL21 (DE3) for recombinant expression and then sequenced for confirmation.

**Table 1:** Primers used for site-directed mutagenesis.

**Primer**	**Sequence (from 5^**′**^ to 3^**′**^ end)**
R105A	F: TTCACAAACGCGAGGGTTTTTGGTCCAATGGG
	R: AAAAACCCTCGCGTTTGTGAAGACAGAGT
R372A	F: ATTGCTGTTGCACTTGATAGGCTCTGTAAGAAGGATG
	R: CCTATCAAGTGCAACAGCAATTGGAAACATTCTGAGAGTC
R440A	F: ACTGGACCCGCAAATTGCATTGGCATGAGGTTTG
	R: AATGCAATTTGCGGGTCCAGTTCCAAAAGGCAGGT

*The underline shows the bases corresponding to the mutant amino acids, all primers were synthesized by Sangon Biotech (Shanghai, China)*.

The protein expression and purification process of bacteria was based on the published article of our laboratory (18). Then, the purified proteins were concentrated through concentrator tubes and confirmed by SDS-PAGE and Western blot. The protein concentration of each sample was determined by the method of Bradford (20).

### The Protein Activity Verification of CYP3A24 and Its Mutants

Testosterone (TS) is a specific substrate of CYP3A, which can generate 6β-OHTS under the action of CYP3A (21). In order to study the protein activity of CYP3A24 and its mutants, we incubated each protein with TS. The experimental steps were as follows: protein (0.5 mg/mL) and NADPH (1 mmol/L) were added to PBS (pH 7.4) for 10 min at 37°C, then TS (Dr. Ehrenstorfer GmbH, Germany, 1 μmol/L) was added to start the reaction. The total reaction volume was 200 μL. At 60 min after drug addition, 200 μL of ice-cold acetonitrile was added to terminate the reaction. After centrifugation at 12,000 g/min for 15 min, the supernatant was filtered through a 0.22 μm membrane and analyzed by LC-MS/MS (AB SCIEX API5000, USA). For TS and 6β-OHTS, chromatographic separation occurred using a Thermo Scientific Hypersil Gold C18 column (150 × 2.1 mm, 5 μm particles), and analytes were detected on an API5000 mass analyser. Mobile phase A was 0.1% formic acid in water, and mobile phase B was methanol. The mass spectrometric parameters for TS and 6β-OHTS was shown in Table 2. The flow rate was 0.2 mL/min with a linear gradient under the following conditions: 0–0.10 min 5–85% B, 0.10–4.10 min 85% B, 4.10–8.00 min 85–5% B. The injection volume was set to 10 μL. The limit of quantitation (LOQ) of the analysis was 0.1 μmol/L, and the limit of detection (LOD) of the analysis was 0.02 μmol/L. All inter-assay coefficients of variation were within 8.6%, the intra-assay coefficients of variation were within 12.4%, and the recovery rates were between 84.5 and 115.4%. After activity verification, CYP3A24 and its mutants were co-incubated with FF and DOX, and the key amino acid residues for metabolism of FF and DOX were studied by the determination of FFA production.

**Table 2:** Optimized characteristic ion mass spectrometry parameters for TS and 6β-OHTS.

**Drug**	**Polarity**	**Precursor ion (m/z)**	**Product ion (m/z)**	**Collision energy (V)**
TS	Positive	289.0	109.0	20
			97.0	35
6-β OH-TS	Positive	305.1	269.3	20
			184.0	25

### Animal Experimental Design

In this study, a total of 54 Boaer-cross goats (*Capra hircus*) of both sexes, about 8 months old, were selected, weighing 28–36 kg. During the 1st week of acclimatization, all animals were raised in the usual way, with free access to feed and water. The experiment was conducted in accordance with the Guidelines for the Care and Use of Laboratory Animals issued by HZAU and approved by the Ethics Committee of Veterinary College of HZAU.

Throughout the experiment, all goats had *ad libitum* access to feed and water without antibiotics. The goats were divided into three groups, namely the control group (6 animals), the single group (24 animals), and the combined group (24 animals). The control group was fed without medication. In the single group, FF (Zhonglongshenli Animal Pharmaceutical Co., Ltd. Hefei, China, specification: 10 mL: 1 g) was injected into the neck muscles at 48 h intervals, twice in total. In the combined group, FF was injected into the neck muscles at 48 h intervals, twice in total, and DOX-hyclate (Zhonglongshenli Animal Pharmaceutical Co., Ltd. Hefei, China, specification: 10 mL: 1 g) was injected into the neck muscles, once a day for 3 days. The volume of the treatment solution was calculated individually for each goat to provide a dose equivalent to 20 mg FF/kg bw and 10 mg DOX/kg bw.

The control group was euthanized by use of ketamine hydrochloride (Fujian Gutian Pharmaceutical Co. Ltd., China: 10–15 mg/kg bw) and xilazine hydrochloride (Shengda Animal Medicine Co. Ltd., China: 2 mg/kg bw) for anesthesia followed by euthanasia with T61 (Bayer Animal Health GmbH, Germany: 4–6 mg/50 kg bw) and tissue samples (liver, kidney and muscle) were collected and were used to the validation of the analytical method for the compounds FF and FFA.

Goats of the single group (treated with FF) and goats of the combined group (treated with FF and DOX) were euthanized as previously described at 0.5 (*n* = 4), 1 (*n* = 4), 3 (*n* = 4), 7 (*n* = 4), 14 (*n* = 4), and 21 days (*n* = 4) after the last dose of 20 mg FF/kg bw (single group) and after the last dose of 20 mg FF/kg bw plus 10 mg DOX/kg bw (combined group). Goats were immediately exsanguinated, and tissue specimens of liver, kidney and muscle were collected separately. Each of the tissue specimens was carefully weighed and stored frozen at −20°C until assayed for concentrations of FF and FFA.

### Gene Expression of CYP3A24 in Goats

Real-time quantitative polymerase chain reaction (qPCR) was used to detect the expression of CYP3A24 in treated goats. Total RNA was extracted from goat liver by using the Trizol method. The concentration and purity of RNA was detected by Q3000 (Thermo, USA). The extracted RNA was reverse transcribed into cDNA. Goat-specific primers have shown as Table 3. The reaction procedure is: stage 1, 95°C for 30 s; stage 2, 40 cycles at 95°C for 3 s, 60°C for 10 s and 72°C for 20 s; stage 3, melt phase. The data were analyzed by the 2^ΔΔ*Ct*^ method.

**Table 3:** Primers sequences of goat CYP3A24 and β-actin for qPCR.

**Gene**	**Primer sequences (5^**′**^ to 3^**′**^)**
CYP3A24	F: ATGCAATTTCGGGGTCCAGT
	R: GGCACCTCCGACCTATGATG
β-actin	F: GGACTTCGAGCAGGAGATGG
	R: CCAGGAAGGAAGGCTGGAAG

*All primers were synthesized by Sangon Biotech (Shanghai, China)*.

### Sample Pre-treatment and Residue Detection

The method of FF and FFA pre-processing in the target tissues was as follows: Tissue samples weighing 1.00 ± 0.01 g were placed in a 10 mL centrifuge tube, then 5 mL 2% ammoniated ethyl acetate was added, and the tube was vortexed, sonicated and centrifuged. The above steps were repeated, and the supernatants were pooled and dried under a stream of nitrogen. The residue was reconstituted in 5% acetic acid in water, vortexed and passed through an MCX solid-phase extraction column (the order of the column was methanol, water, sample, 5% acetic acid water and 8% ammoniated ethyl acetate), then the eluate was dried under nitrogen, re-dissolved in 2 mL of degreased n-hexane, passed through a 0.22 μm filter membrane and subjected to LC-MS/MS (AB SCIEX API5000, USA) analysis.

**Figure 1:**
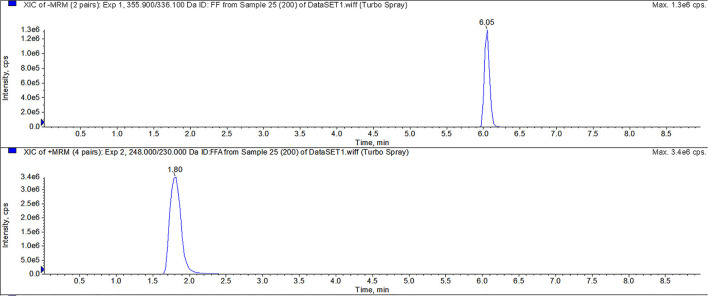
LC-MS/MS method for detecting FF, and FFA. The peak times of FFA and FF were 1.80 and 6.05 min, respectively.

For FF and FFA, chromatographic separation was performed using a Thermo Scientific Hypersil Gold C18 column (150 × 2.1 mm, 5 μm particles). Mobile phase A was 0.1% formic acid in water, and mobile phase B was acetonitrile. The mass spectrometric parameters for FF and FFA was shown in Table 4. The flow rate was 0.3 mL/min with a linear gradient under the following conditions: 0–1 min 10% B, 1–5 min 10–70% B, 5–6 min 70% B, 6–6.1 min 70–10% B, and 6.10–9.00 min 10% B. All calibration curves exhibited a correlation co-efficient (*r*) exceeding 0.99 across the concentration range. All inter-assay coefficients of variation in each tissue were within 9.93%, the intra-assay coefficients of variation in each tissue were within 11.08%, and the recovery rates in each tissue were between 70.62 and 113.55%. The LC-MS/MS results of FF and FFA standard products showed that the method had good specificity. For FF and FFA, the LOQ of the analysis was 10 μg/kg, and the LOD of the analysis was 2 μg/kg. The peak times of FFA and FF were 1.80 and 6.05, respectively (Figure 1).

**Table 4:** Optimized characteristic ion mass spectrometry parameters for FF and FFA.

**Drug**	**Polarity**	**Precursor ion**	**Product ion**	**Collision energy**
FF	Positive	355.9	336.1	22
			118.8	35
FFA	Positive	247.9	229.7	18
			130.1	34

### Analysis of Experimental Data

Significance analysis between different groups using one-way ANOVA with SPSS Statistics, version 18.0 (SPSS Inc., Chicago, IL). 0.01 < *P* < 0.05 indicates a significant difference, marked with ^*^; *P* < 0.01 indicates a very significant difference, marked with ^**^.

The withdrawal period was estimated by linear regression analysis of log-transformed tissue concentrations and was determined at the time when the 95% upper one-side tolerance limit was below the MRL with 95% confidence (22).

## Results

### CYP3A24 May Be the Main Metabolic Enzyme of FF in Breed Goat Liver Microsomes

**Figure 2:**
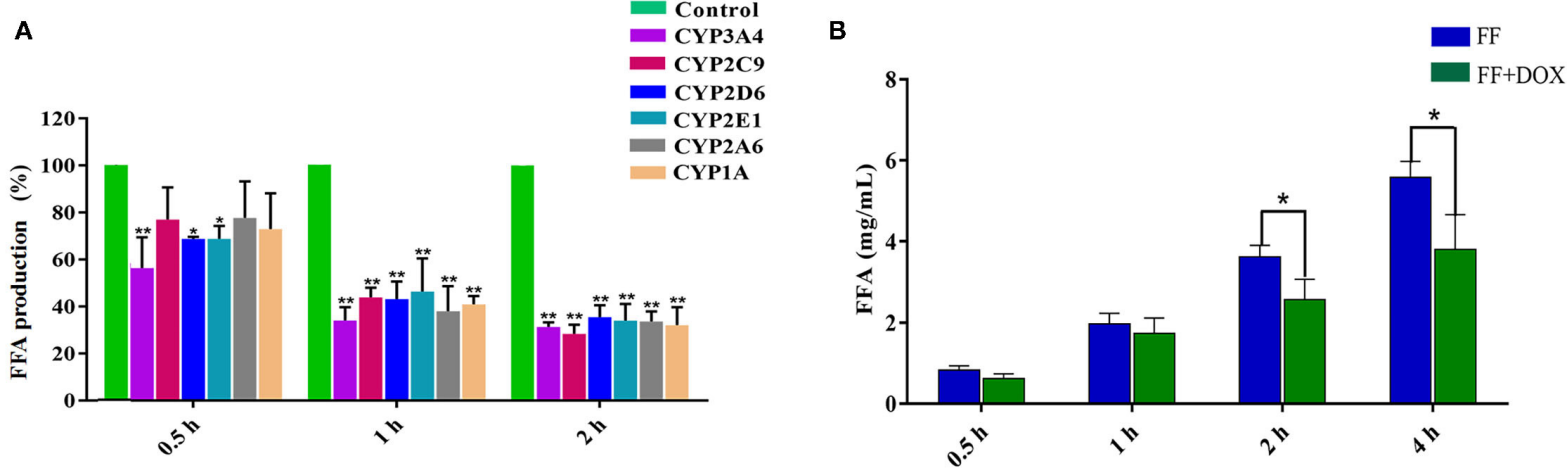
The influence of specific inhibitors of CYP450 [ketoconazole (CYP3A4), diosmetin (CYP1A), quinidine (CYP2D6), fomepizole (CYP2E1), methoxsalen (CYP2A6) and sulfaphenazole (CYP2C9)] on the generation of FFA **(A)**. The effect of DOX on the metabolism of FF in goat liver microsomes **(B)**. **(A)** The horizontal axis represents the incubation time, and the vertical axis represents the FFA production in the inhibitor group to that in the control group. **(B)** The horizontal axis represents the incubation time of the drug and goat liver microsomes, and the vertical axis represents the amount of FFA produced. The results were expressed as mean ± standard deviation (error bars) and repeated three times of each reaction. *P* < 0.05 (*) was considered to be statistically significant. *P* < 0.01 (**) was considered a significant difference.

The influence of CYP450-specific inhibitors on the production of FFA is shown in Figure 2A. Compared with the inhibitor-free group (control group), the production of FFA decreased at all times after the addition of CYP450 inhibitors. The inhibition percentage of FFA production was as follows: at 0.5 h of incubation, the order was CYP3A4 (43.66%) > CYP2E1 (31.20%) > CYP2D6 (31.19%) > CYP1A (27.13%) > CYP2C9 (23.01%) > CYP2A6 (22.28%) inhibitors. After 1 h of incubation, the order of influence on FF metabolism was: CYP3A4 (65.94%) > CYP2A6 (62.02%) > CYP1A (59.13%) > CYP2D6 (56.90%) > CYP2C9 (56.16%) > CYP2E1 (53.71%) inhibitors. After 2 h of incubation, the order of influence on FF metabolism was: CYP2C9 (71.70%) > CYP3A4 (68.73%) > CYP1A (67.95%) > CYP2A6 (66.44%) > CYP2E (66.06%) > CYP2D6 (64.62%) inhibitors. In general, the result showed that CYP3A is the most critical enzyme in FF metabolism.

### In Breed Goat Liver Microsomes, DOX Slows Down FF Metabolism

The result is represented in Figure 2B. After 0.5 and 1 h incubations, there was no significant difference in the amount of FFA produced between the single group (FF) and the combined group (FF + DOX). After 2 and 4 h incubations, the production of FFA in the single group was significantly higher (41.4 and 46.5%, respectively), than that of the combined group. It is speculated that FF and DOX interact in liver microsomes, slowing down FF metabolism.

### Homologous Modeling Molecular Docking

**Figure 3:**
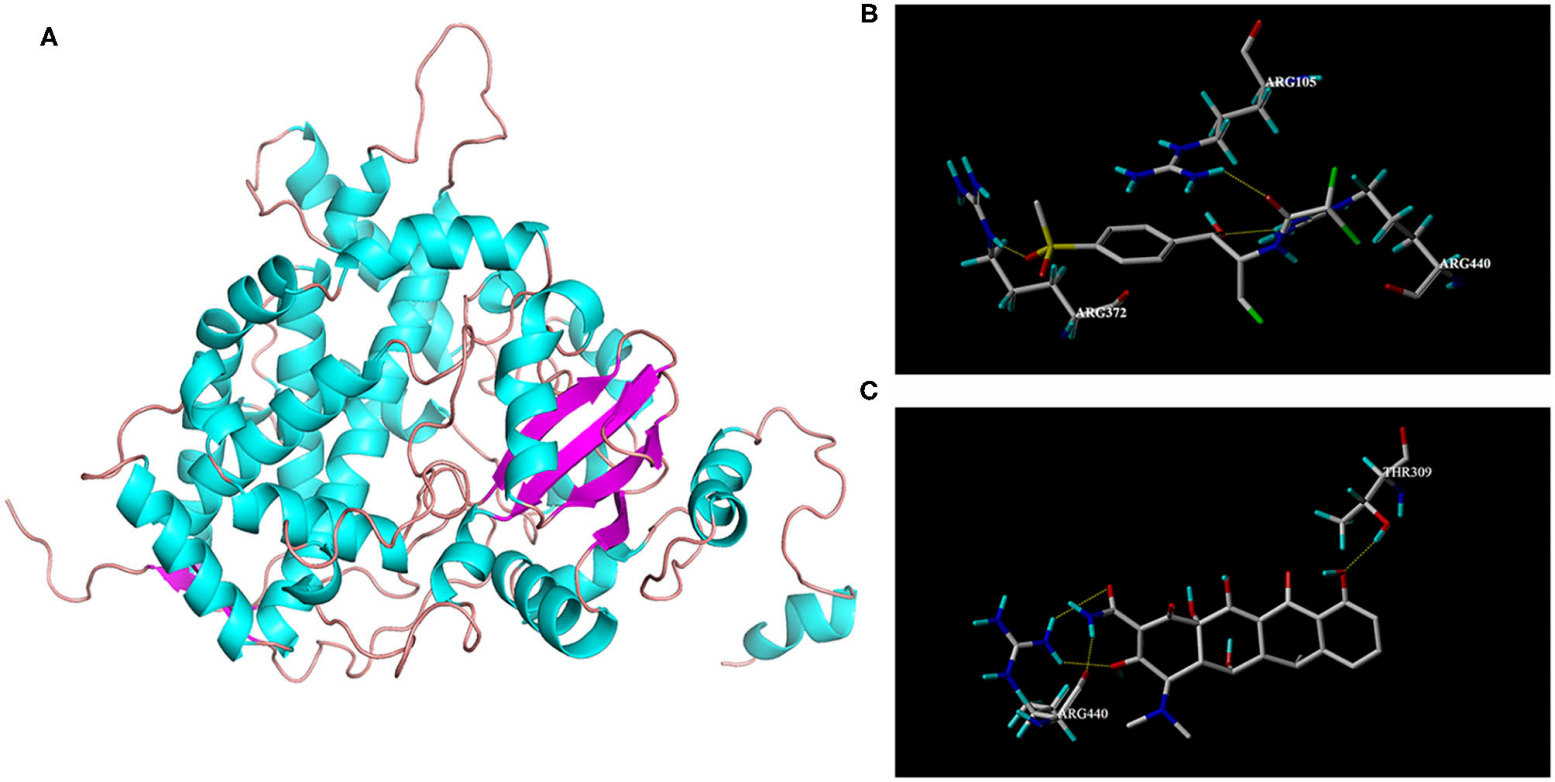
3D structure of CYP3A24 **(A)** and interaction with FF **(B)** and DOX **(C)**, respectively. **(A)** 3D structure of CYP3A24 was represented as a cartoon, with α-helices colored in cyan, β-stands in magenta, and loops in salmon. **(B)** Amino acid residues involved in the interaction of CYP3A24 with FF. **(C)** Amino acid residues involved in the interaction of CYP3A24 with DOX. The hydrogen bonds were represented as yellow dotted lines. The FF molecular was colored in gray.

The protein structure of goat CYP3A24 was modeled using CYP3A4 as a template, which is displayed by the visualization software Pymol, as shown in Figure 3A. In order to better explore the mechanism by which DOX affects FF metabolism, FF and DOX were molecularly docked with CYP3A24, and the docking results are shown in Figures 3B,C. For FF, three hydrogen bonds were formed between the CYP3A24 protein and FF in the active pocket. Among the hydrogen bonds forming AAs, one hydrogen bond was, respectively, formed with R105, R372, R440. The key amino acid residues in CYP3A24 that play a role in FF metabolism may be R105, R372, and R440. For DOX, four hydrogen bonds were formed between the CYP3A24 protein and DOX in the active pocket. Among the hydrogen bonds forming AAs, in which one hydrogen bond was formed with T309, and three hydrogen bonds were formed with R440. The amino acid residues in CYP3A24 that interact with DOX may be T309 and R440. It is speculated that the interaction between FF and DOX may be related to R440.

### Protein Expression of CYP3A24 and Its Mutants

**Figure 4:**
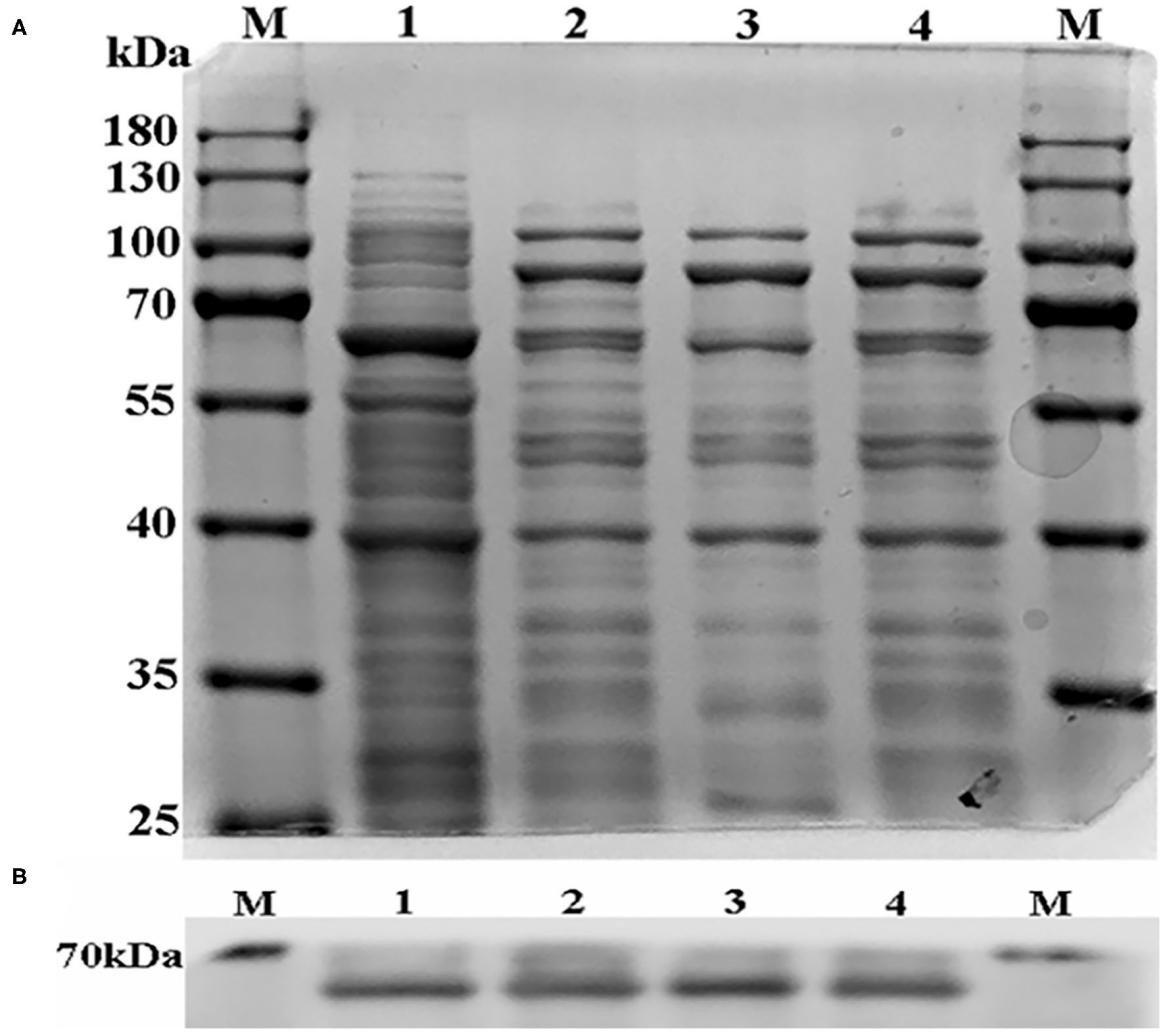
The SDS-PAGE result of CYP3A24 and its mutants **(A)**. The Western blot result of CYP3A24 and its mutants **(B)**. **(A)** The sample volume in each line was 20 μL, and no protein quantization was carried out. **(B)** The sample of each protein was 30 μ4g. Line M is protein marker, lines 1–4 are CYP3A24, R105A, R372A, R440A, respectively.

The R (arginine) side chain is positively charged and forms ionic interactions with negatively charged groups. A (alanine) has a non-polar side chain, which is non-reactive and can eliminate the ionic interaction (23). For this reason, we mutated R to A. According to the website http://web.expasy.org/compute_pi/, the protein molecular weight of CYP3A24 is predicted to be about 58 kDa. As shown in Figure 4, the results of SDS-PAGE and Western blot show that the molecular weight of the protein is consistent with the prediction.

### CYP3A24 Enzyme and Its a Mutant Protein Had Good Activity

**Figure 5:**
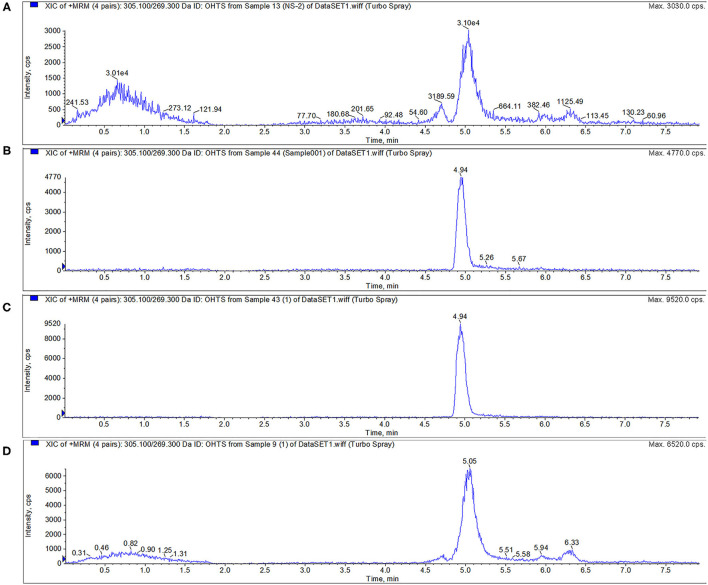
Activity verification of CYP3A24 and mutant protein. The results **(A–D)** show that CYP3A24, R105A, R372A and R440A could metabolize TS into 6β-OHTS. Note: **(A–D)** are the chromatograms of CYP3A24 **(A)**, R105A **(B)**, R372A **(C)**, and R440A **(D)**.

TS can be metabolized into 6β-OHTS by CYP3A enzyme. The peak time of 6β-OHTS was about 4.95 min. The results show that CYP3A24, R105A, R372A, and R440A could metabolize TS, and all the proteins were active (Figure 5).

### R440A May Be a Key Amino Acid Through Which DOX Affects the FF Metabolism

**Figure 6:**
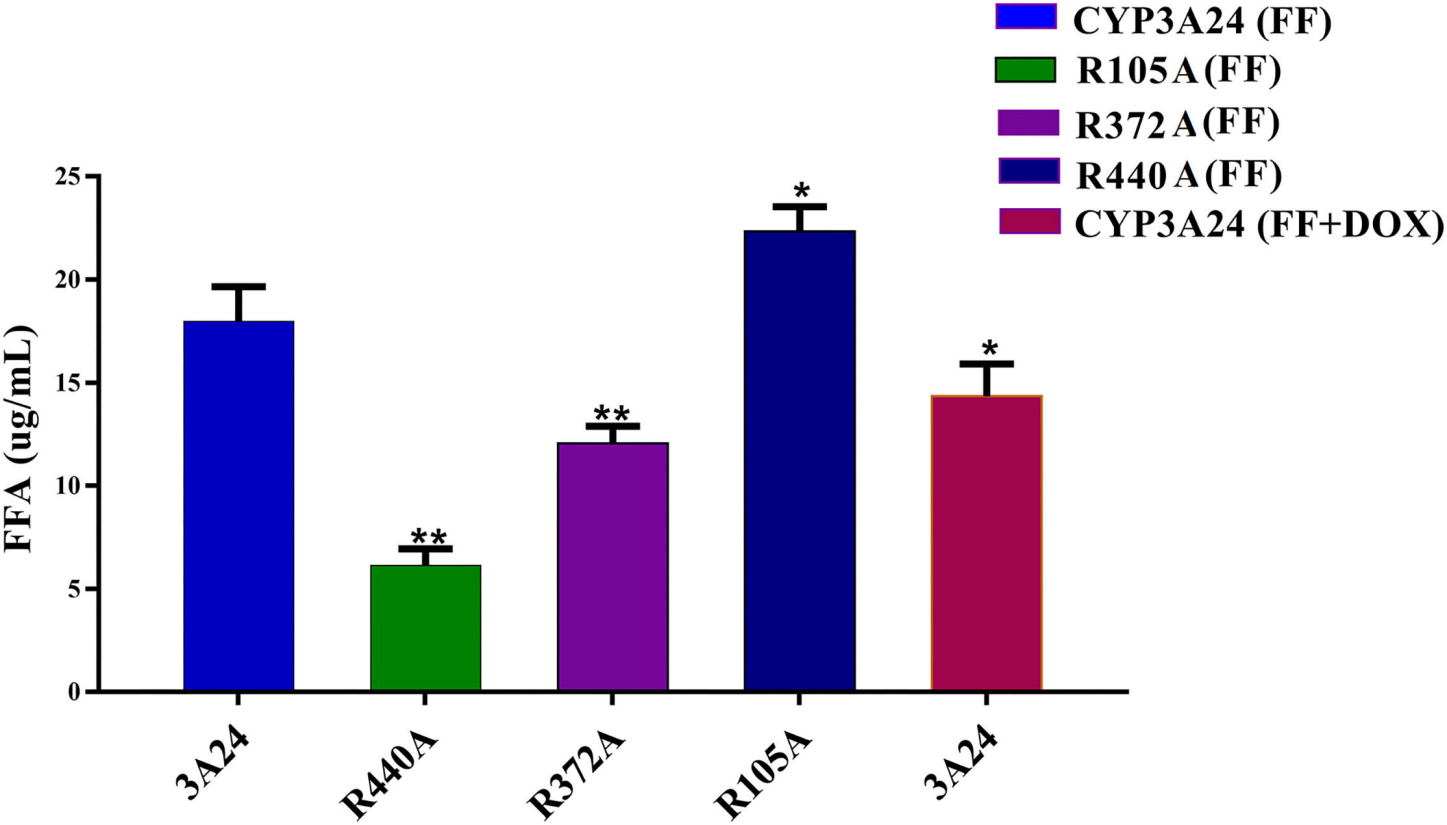
Incubation of CYP3A24 with FF and DOX and its mutants (R105A, R372A, and R440A). The vertical axis represents FFA production. The data were expressed as mean ± standard deviation (error bars) (*n* = 3). **(*P* < 0.01) and *(*P* < 0.05) indicate statistically significant difference between the CYP3A24 and each mutant.

According to Figure 6 when incubated with CYP3A24, the addition of DOX reduced the production of FFA in 17.8%, that is, the metabolism of FF was inhibited. The key amino acid sequence that reduced FF metabolism was: R440A (65.8%) and R372A (32.9%). R440A is a key amino acid for CYP3A24 enzyme to metabolize FF. Since R440A is the common site in the docking result of FF and DOX, it is speculated that the reason why DOX inhibits FF metabolism is that DOX competes with R440 for the CYP3A24 enzyme.

### The Effect of DOX and FF on CYP3A24 Gene Expression in Goats

**Figure 7:**
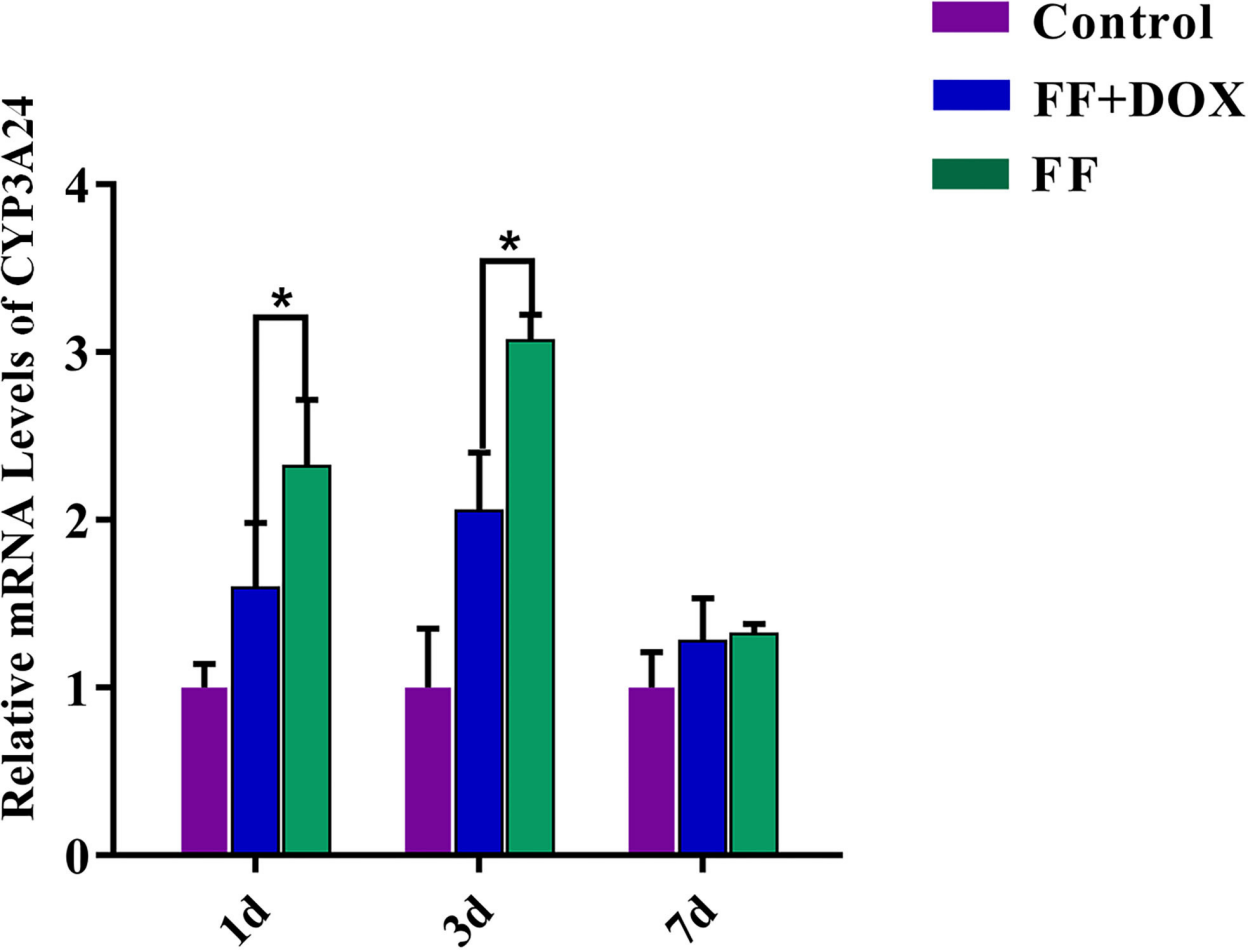
CYP3A24 gene expression in goats. The data were expressed as mean ± standard deviation (error bars) (*n* = 3). *(*P* < 0.05) indicates statistically significant difference between the combined group (FF + DOX) and the single group (FF).

The concentration and purity of RNA extracted from goat liver were good. The A260/280 ratio of all RNA extracted was between 1.8 and 2.0, and the concentration was about 2,000 ng/μL. The effect of single administration of FF and co-administration of FF and DOX on the expression level of the CYP3A24 gene in goats is shown in Figure 7.

When FF was stopped for 1 and 3 days, the gene expression level of CYP3A24 enzyme was up-regulated in the single groups, about 2.3 and 3-fold, respectively compared to control.

When FF + DOX was stopped for 1 and 3 days, the up-regulation of CYP3A24 enzyme gene expression in the combined groups decreased significantly compared to single groups, about 0.7 and 1-fold, respectively. This indicates that DOX can inhibit up-regulation of the CYP3A24 gene.

### Tissue Residue Depletion

Residues of FF and its metabolite FFA in tissue specimens after intramuscular administration of FF (20 mg/kg bw, at 48 h intervals, twice in total) in single group and after FF (20 mg/kg bw, at 48 h intervals, twice in total) plus DOX (10 mg/kg bw, once a day for 3 days) in combined group, were determined. The tissue concentration-time profiles are presented in Table 5 for liver, kidney, and muscle from single and combined groups at 0.5, 1, 3, 7, 14, and 21 days after administration of the final dose of FF (single group) and a after administration of the final dose of FF plus DOX (combined group).

**Table 5:** Tissue concentrations of FF and FFA for goats treated intramuscularly with FF (single group) and for goats treated with FF plus DOX (combined group).

**Tissue**	**Group** **Single: 20 mg FF/kg bw, at 48 h intervals, twice in total**	**Time after last dose (days)**	**FF (μg/kg)**	**FFA (μg/kg)**	**FF+FFA (μg/kg)**
	**Combined: 20 mg FF/kg bw, at 48 h intervals, twice in total plus 10 mg DOX/kg bw, once a day for 3 days**				
Liver	Single	0.5	2791.1 ± 256.7	1715.0 ± 284.7	4618.5 ± 188.0
		1	2053.8 ± 310.5	1324.0 ± 234.8	3377.8 ± 155.6
		3	936.2 ± 148.3	827.0 ± 136.8	1763.2 ± 76.1
		7	338.4 ± 126.3	678.2 ± 77.1	1005.4 ± 67.8
		14	178.7 ± 26.5	294.1 ± 41.6	472.8 ± 46.0
		21	44.5 ± 17.6	88.5 ± 26.6	133.0 ± 41.8
	Combined	0.5	2904.7 ±195.7	1687.5 ± 305.5	4592.2 ± 480.0
		1	2181.0 ± 189.1	1264.1 ± 133.5	3445.1 ± 173.6
		3	937.1 ± 193.3	858.0 ± 93.7	1795.1 ± 173.6
		7	342.4 ± 69.9	630.4 ± 110.5	972.8 ± 52.1
		14	204.4 ± 52.1	329.2 ± 52.4	533.5 ± 33.0
		21	62.6 ± 19.5	113.0 ± 15.3	175.6 ± 6.8
Kidney	Single	0.5	2259.8 ± 203.0	1297.1 ± 239.3	3556.9 ± 209.3
		1	1772.2 ± 141.8	1047.9 ± 105.4	2820.1 ± 177.4
		3	843.1 ± 123.7	677.1 ± 88.7	1520.2 ± 70.2
		7	327.4 ± 43.4	328.5 ± 49.7	656.0 ± 24.2
		14	130.7 ± 17.1	168.6 ± 33.3	299.3 ± 26.1
		21	37.5 ± 8.6	62.0 ± 19.9	99.5 ± 27.6
	Combined	0.5	2373.0 ± 250.9	1194.3 ± 124.9	3567.3 ± 201.8
		1	1873.8 ± 164.4	977.7 ± 216.4	2851.5 ± 178.3
		3	812.2 ± 117.8	687.1 ± 60.2	1499.4 ± 66.0
		7	369.3 ± 69.7	315.8 ± 88.2	685.1 ± 61.5
		14	140.1 ± 46.8	164.8 ± 58.6	304.9 ± 14.0
		21	50.7 ± 8.1	83.0 ± 20.9	133.7 ± 19.6
Muscle	Single	0.5	1762.3 ± 201.0	1083.7 ± 101.3	2846.1 ± 301.9
		1	858.1 ± 43.2	492.6 ± 49.2	1350.7 ± 64.0
		3	376.2 ± 58.1	322.7 ± 72.8	698.9 ± 35.6
		7	162.1 ± 4.6	107.5 ± 11.9	269.6 ± 16.4
		14	48.6 ± 7.6	68.1 ± 13.4	116.7 ± 20.9
		21	ND	ND	ND
	Combined	0.5	1701.1 ± 111.2	862.4 ± 103.4	2563.5 ± 212.1
		1	920.5 ± 69.2	479.2 ± 29.8	1399.6 ± 69.7
		3	420.5 ± 31.8	265.6 ± 65.7	686.1 ± 50.1
		7	170.0 ± 23.6	97.0 ± 25.3	267.1 ± 7.9
		14	50.6 ± 3.7	62.3 ± 5.7	112.9 ± 8.9
		21	ND	ND	ND

*Each value is the mean ± SD for 4 goats*.

### Withdrawal Period Estimation

**Figure 8:**
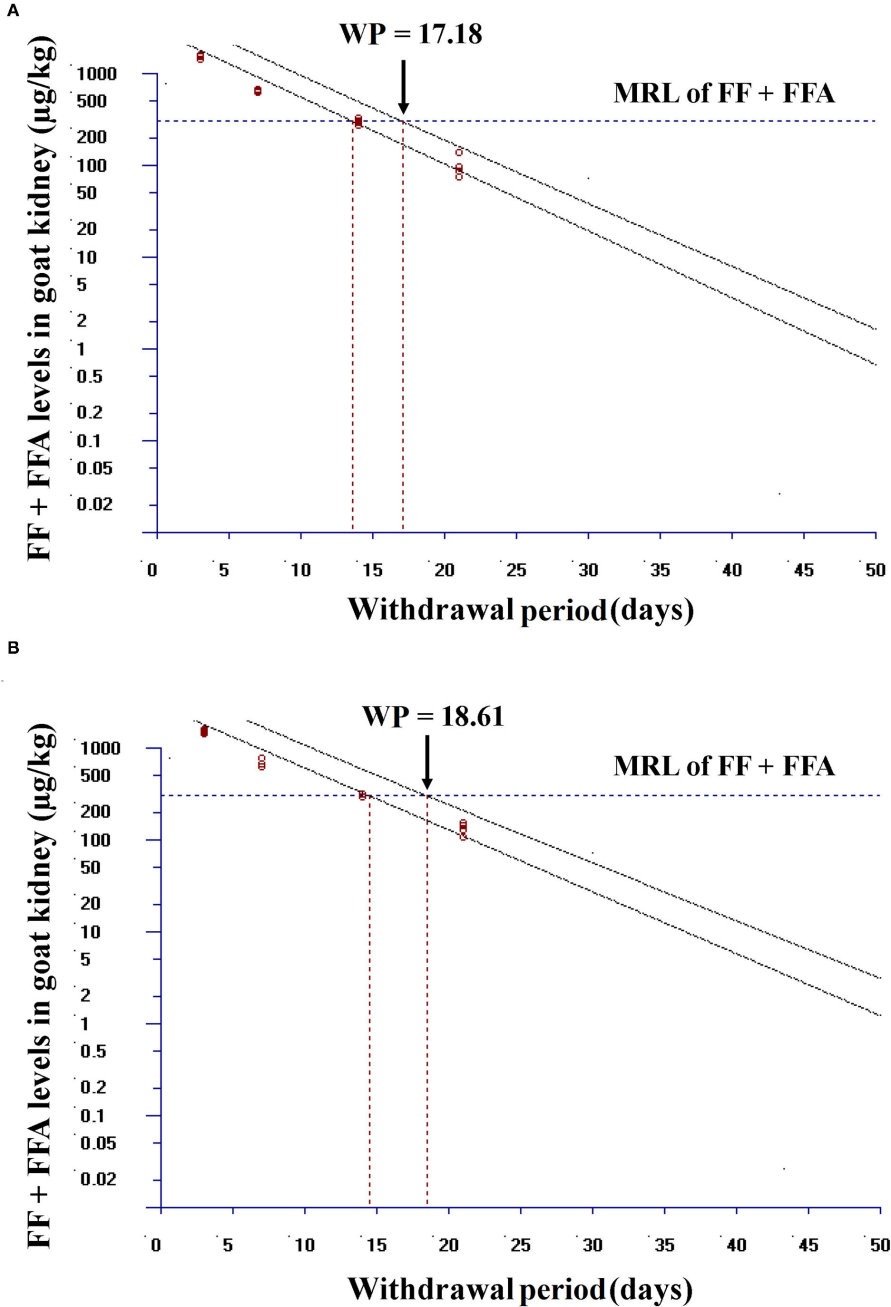
Plot of the withdrawal period calculation for FF in goat kidney at the time when the one-sided 95% upper tolerance limit is below the EU MRL for FF (300 μg/kg). **(A)** In single group, after intramuscular administration of FF (20 mg FF/kg bw, at 48 h intervals, twice in total) and **(B)** in combined group, after intramuscular administration of FF (20 mg FF/kg bw, at 48 h intervals, twice in total) plus DOX (10 mg DOX/kg bw, once a day for 3 days). Residue marker is the sum of florfenicol (FF) and its metabolite florfenicol-amine (FFA).

Linear regression analysis of the logarithmic transformed data can be considered for the calculation of the withdrawal periods. Using this approach, the withdrawal period was determined as the period when the one-sided, 95% upper tolerance limit of the regression line with 95% confidence level was below the MRL (22). Using this approach and considering the marker residue for the MRL (the sum of FF and its metabolite FFA) recommended by de EU, the withdrawal period for FF was calculated for single group and combined group in kidney (tissue which showed slower residue depletion). After intramuscular administration of FF (20 mg/kg bw, at 48 h intervals, twice in total) in single group and after FF (20 mg/kg bw, at 48 h intervals, twice in total) plus DOX (10 mg/kg bw, once a day for 3 days) in combined group, the withdrawal periods were 17.18 days and 18.61 days, respectively (Figures 8A,B). When DOX is used in combination with FF, the withdrawal period of FF in the kidney was extended by 1 day.

## Discussion

In veterinary medicine, drugs are often used in combination; improving efficacy is the primary factor in drug combination. However, DDI often produces some adverse effects (24). Metabolism is a key factor affecting DDI, and CYP450 enzymes are a key factor affecting metabolism (25). The inhibition of CYP enzymes may cause the accumulation of drugs in the body, resulting in toxicity or prolonging the drug withdrawal period (26). Therefore, it is necessary to study combinations of drugs from the perspective of enzymes. FF and DOX are often used together in clinical treatment. Previous studies have shown that the metabolism of FF in rabbits and chickens is affected by CYP3A (11, 12), and DOX has the effect of inhibiting CYP3A (13). Therefore, this research studies the combined use of DOX and FF from the perspective of liver microsomes and recombinant metabolic enzymes. Moreover, *in vivo* experiments were also conducted to verify the effects of the two antibacterial on metabolism, and to recommend a withdrawal period, so as to better guide clinical medication.

*In vitro* goat liver microsome experiments showed that CYP3A is the most important metabolic enzyme that affects the metabolism of FF (Figure 2A). This is consistent with studies in rabbit and chicken (11, 12). The addition of DOX will slow down the metabolism of FF (Figure 2B). If the functionally homologous proteins have sequence homology >30%, then a known protein crystal structure can be used as a template to establish a highly accurate target protein structure model. The sequence homology between goat CYP3A24 and human CYP3A4 enzymes reached 76.5%. So we used human CYP3A4 as a template for homology modeling and obtained CYP3A24 (Figure 3A). Then we have molecularly docked FF and DOX with CYP3A24 enzyme and screened out the possible key amino acids for FF metabolism as R105, R372, and R440 (Figure 3B), and T309 and R440 as possible key amino acids for DOX metabolism (Figure 3C). Site-directed mutagenesis is often used to study the impact of specific sites on the overall structure (27). Therefore, in this experiment, the three amino acids (R105, R372, and R440) were mutated to R105A, R372A, and R440A. The mutated protein was incubated with the drug to determine that R440A may be the key amino acid through which DOX affects FF (Figure 6). Combined with previous research on the interaction between R440 and CYP2D6, R440 is very likely to be the main site of CYP2D6 binding to reductase (28). This research suggests that R440 plays an important role in the electron transfer and binding between CYP3A24 and its reductase. When it is destroyed, the metabolism of FF and DOX will be affected; when FF is used in combination with DOX, the DOX will compete for R440 on the CYP3A24 enzyme, which will slow down FF metabolism.

Notably, the effect of DOX on FF metabolism was significant. *In vivo* experiments have shown that DOX can inhibit the up-regulation of CYP3A24 gene expression caused by FF (Figure 7). Residue depletion studies in goats showed that the addition of DOX would slow down the elimination half-life of FF and FFA (Table 3). EMA withdrawal-period calculation-program WT1.4 software (22) was used to analyse the withdrawal period of FF from goat target tissue concentrations of FF and FFA (Figure 8). Notably, the effect of DOX on FF metabolism was significant. The combination of FF and DOX caused a prolonged withdrawal period of FF in the kidney; the withdrawal period of FF in the kidney was extended by 1 day. In addition, the kidney toxicity of FF has been confirmed in animals. FF can up-regulate the expression of pro-apoptotic factors and accelerate the abnormal apoptosis of renal cells (29). Therefore, whether the effect of DOX on FF metabolism in the kidney will lead to the enhancement of this toxic effect remains to be studied.

## Data Availability Statement

The raw data supporting the conclusions of this article will be made available by the authors, without undue reservation.

## Ethics Statement

The animal study was reviewed and approved by Ethics Committee of Veterinary College of Huazhong Agricultural University (HZAU).

## Author Contributions

XW and YY: software, validation, formal analysis, investigation, data curation, visualization, and writing—original draft. M-AM, MM, BL-T, M-RM-L, and IA: software, validation, formal analysis, investigation, data curation, visualization, writing—original draft, and writing—review and editing. XW and AA: conceptualization, methodology, resources, investigation, writing—original draft, writing—review and editing, supervision, and project administration. All authors contributed to the article and approved the submitted version.

## Funding

This work was supported by the National Key Research and Development Program of China (2018YFC1603005), the Fundamental Research Funds for the Central Universities (2662020DKPY020), and Project Ref. PID 2020-115979RR-C33 from the Ministerio de Ciencia e Innovación, Spain.

## Conflict of Interest

The authors declare that the research was conducted in the absence of any commercial or financial relationships that could be construed as a potential conflict of interest.

## Publisher's Note

All claims expressed in this article are solely those of the authors and do not necessarily represent those of their affiliated organizations, or those of the publisher, the editors and the reviewers. Any product that may be evaluated in this article, or claim that may be made by its manufacturer, is not guaranteed or endorsed by the publisher.
